# Effects of White Sorghum Flour Levels on Physicochemical and Sensory Characteristics of Gluten-Free Bread

**DOI:** 10.3390/foods12224113

**Published:** 2023-11-13

**Authors:** Fahrunnisa Adzqia, Suntaree Suwonsichon, Masubon Thongngam

**Affiliations:** 1 Department of Product Development, Faculty of Agro-Industry, Kasetsart University, Bangkok 10900, Thailand; fahrunnisa.a@ku.th; 2 Kasetsart University Sensory and Consumer Research Center (KUSCR), Department of Product Development, Faculty of Agro-Industry, Kasetsart University, Bangkok 10900, Thailand; 3 Department of Food Science and Technology, Faculty of Agro-Industry, Kasetsart University, Bangkok 10900, Thailand; masubon.t@ku.th

**Keywords:** gluten-free bread, gluten-free flour, sorghum, descriptive analysis, flavor, texture, wheat substitute, wheat alternative, bakery

## Abstract

This research studied the effects of white sorghum flour levels at 0, 10, 25, 40, 70, 85 and 100% in the matrix of rice and tapioca flours and corn starch on the properties of flour blends and the qualities of gluten-free (GF) bread. Single and composite flours were analyzed for moisture content, color and pasting properties. GF bread samples prepared from composite flours were analyzed for specific volume, moisture content, water activity, crumb color and instrumental texture. Sensory profiles of the breads were determined by nine trained descriptive panelists. The results show that increasing the sorghum flour content increased (*p* ≤ 0.05) color intensity, pasting temperature and setback viscosity, while it decreased (*p* ≤ 0.05) the peak and breakdown viscosities of flour blends. For GF bread, increasing white sorghum flour levels in the blends primarily affected specific volume, color, flavor and texture characteristics, leading to decreases (*p* ≤ 0.05) in specific volume, cohesiveness, springiness, chewiness and moistness, but increases (*p* ≤ 0.05) in color intensity, brown and nutty flavors, graininess and roughness. White sorghum flour could be used in the blends at the maximum level of 25% to get a good bread volume without sacrificing texture quality.

## 1. Introduction

Bread is one of the most widely consumed foods worldwide due to its nutritional quality, convenience and palatability [1]. Wheat flour is conventionally used for breadmaking as it contains gluten protein, which is responsible for the physical and functional properties of bread dough [2]. However, about 1% of the world population suffers from gluten intolerance or celiac disease (CD), a chronic digestive and immune disorder that damages the gastrointestinal system [3]. The only accepted treatment for CD is a life-long adherence to a gluten-free (GF) diet [4]. This situation leads to the development of GF bread using alternative natural materials such as local grains, legumes and tubers. Currently, the GF diet is also popular among people who have not been diagnosed with CD [2]. The GF bakery market is forecasted to grow at a 9.8% annual rate during the period 2023–2028, with the fastest growth rate in the Asia Pacific region [5].

Studies have been conducted to promote the utilization of GF flours or starches from locally grown crops such as sweet potato [2], corn [6,7,8], cassava [2,6,9], potato [6,9], millet [10], rice [6,7,8,9,11], buckwheat [7], teff [7,8], amaranth [8], quinoa [8], tapioca [12] and cowpea [12] in bread. Aside from these crops, sorghum (*Sorghum bicolor* L. Moench) has recently received increasing attention for its GF nature [13]. Sorghum flour is a rich source of nutrients, including proteins, vitamins, minerals and antioxidants, making it a highly nutritious option for GF products [14]. Moreover, research has demonstrated that sorghum-based foods, such as bread, exhibit a lower glycemic index when compared to those made with wheat or rice [15]. In addition to its nutritional benefits, the incorporation of sorghum into human diets contributes to sustainable agricultural practices and global food security [16]. Sorghum is a robust crop capable of thriving in diverse climates, offering a means to diversify food sources and reduce reliance on a limited range of grains [14]. As a worldwide cultivated cereal, sorghum holds great potential as a valuable wheat substitute for breadmaking [2,9,10,16].

Hager and Arendt [7] and Siddeeg et al. [17] investigated the potential use of sorghum as a wheat substitute in bread. Unlike wheat dough, sorghum dough lacked the consistency and elasticity to retain the fermentation gases produced during proofing and baking. Thus, GF bread made with 100% sorghum flour often had low loaf volume and poor crumb properties. Different approaches have been investigated to improve the qualities of sorghum bread, one of which is increasing the starch content of the dough by combining sorghum flour with other flours/starches, such as rice starch and tapioca starch (a starch extracted from storage roots of cassava plant, *Manihot esculenta* Crantz) to facilitate the development of a cohesive crumb network that traps gas bubbles and prevents crust collapse [6]. Akin and Miller [18] observed that bread made with 90% sorghum flour and 10% potato, rice, or tapioca starch, along with the addition of xanthan gum or hydroxypropyl methyl cellulose (HPMC), had a higher loaf volume and better crumb properties than that made with 100% sorghum flour. According to Monthe et al. [2], GF bread formulated with 75% fermented cassava flour, 20% sweet potato flour and 5% sorghum flour exhibited optimal textural properties. In another study, composite flour formulation consisting of 67.18% sorghum flour, 17.82% rice flour and 15% millet flour yielded GF bread with a desirable specific volume, crumb structure, hardness and sensory quality [19].

Depending on the color of grain pericarp and endosperm, sorghum can be classified as white, yellow, brown, red or black. White sorghum flour is considered a favorable substitute for wheat flour in breadmaking due to its neutral color and flavor, as well as its highly resistant starch content [20,21,22,23]. In addition, white sorghum typically contains lower tannin [24] and higher protein contents [25] than red or other colored sorghum varieties. Research by De Aguiar et al. [13] found that bread made with white sorghum flour had a milder and less bitter aftertaste as compared to that made with bronze or brown sorghum flour.

The current research used white sorghum flour in combination with rice and tapioca flours and corn starch for the preparation of GF bread. Based on the authors’ knowledge, such a flour/starch combination has not been studied before. The specific objective of this research was to determine the effects of sorghum flour levels in the blends of rice and tapioca flours and corn starch on the physicochemical properties of flour blends, as well as their effects on the physicochemical and sensory properties of GF bread. The findings from this research should offer new GF flour formulations, and should play an important role in promoting the utilization of sorghum flour in GF products.

## 2. Materials and Methods

### 2.1. Materials

White sorghum grains of KU 804 cultivar were provided by the National Corn and Sorghum Research Center, Kasetsart University, Nakhon Ratchasima, Thailand. The grains were sorted, washed, dried in a tray dryer (Owner Foods Machinery Co., Ltd., Bangkok, Thailand) at 80 °C for 4 h, and then ground using a stainless-steel blender (LX-10A, Shanghai Jiangxin Technology Co., Ltd., Shanghai, China). The obtained whole sorghum grain flour was sieved through a stainless steel 80-mesh screen to get a particle size of 180 µm or less. According to the available literature, the KU804 white sorghum flour contained 9.47% protein, 0.32% lipid, 0.39% ash and 0.33% fiber [14]. 

Rice flour (Varavoot Industry Co., Ltd., Angthong, Thailand), tapioca flour (Five Star Fish E.T.C International Trading Co., Ltd., Nonthanburi, Thailand) and corn starch (Continental Food Co., Ltd., Bangkok, Thailand) were purchased from supermarkets. Pure refined sugar (Mitr Phol Sugar Corp. Ltd., Suphanburi, Thailand), salt (Thai Refined Salt Co., Ltd., Nakhon Ratchasima, Thailand), instant dried yeast (Lessafre Yeast Corp., Marcq-en-Baroeul, France), baking powder (Continental Food Co., Ltd., Bangkok, Thailand), xanthan gum (TR Foods and Bakery L. P., Samut Sakhon, Thailand), calcium propionate (Best Odour Co., Ltd., Bangkok, Thailand), unsalted butter (Thai Dairy Industry Co., Ltd., Phra Nakhon Si Ayutthaya, Thailand) and eggs were acquired from supermarkets or bakery supply stores.

### 2.2. Composite Flour Treatments

Sorghum, rice and tapioca flours and corn starch were mixed at various proportions to form six GF composite flour treatments (T1–T6), as shown in Table 1. The composite flour samples contained 0–85% sorghum flour and 5–33.3% of each of the other flours/starch. As the proportions of sorghum flour increased, those of the other flours/starch decreased such that the sum of all components was 100%. The proportions of rice flour, tapioca flour and corn starch were kept constant at 1:1:1 for all composite flour samples. Sorghum flour (100%) was also included as a treatment (T7) in the study.

### 2.3. Flour Analyses

Flour samples, both single and composite flours, were analyzed for moisture content, as well as color and pasting properties. Moisture content, an indicator of flour stability during storage, was determined according to the AOAC method 10.1.2 [26]. Color values (CIE L*, a* and b*) were measured by a colorimeter (Ultrascan, Hunterlab, Reston, VA, USA) at a 10° observer angle using D65 as a light source. The pasting characteristics were analyzed using Rapid Visco Analyzer (RVA) (Newport Scientific, Warriewood, Australia) according to the AACC method 76–21.01 [27]. Pasting temperature (°C), peak time (min), peak viscosity (cp), breakdown viscosity (cp), set back viscosity (cp) and final viscosity (cp) were recorded. All measurements were performed in triplicate.

### 2.4. Breadmaking Process

Six composite flour samples (T1–T6) and pure sorghum flour (T7) as shown in Table 1 were used for breadmaking. The bread formulation (each batch) consisted of flour (150 g), pure refined sugar (17.54 g), salt (4.28 g), instant dried yeast (3.40 g), baking powder (5.33 g), xanthan gum (3.44 g), calcium propionate (0.45 g), unsalted butter (11.03 g), egg (50.60 g) and warm water (102.97 g). The amount of water added was the same for all treatments. A straight dough method was used to prepare the plain open-top loaf bread samples. All dry ingredients (flour, salt, baking powder, xanthan gum and calcium propionate) were mixed. Sugar and instant dried yeast were dissolved in warm water (about 40 °C) for 5 min. The beaten egg and melted butter were mixed and poured into the mixed dry ingredients, then the yeast mixture was added. Subsequently, all ingredients were mixed using a standing mixer (multipurpose mixer EKM3437W, Electrolux, Stockholm, Sweden) at low speed for 30 s and continued at medium speed for 7 min. The dough (300 g) was put in a greased bread pan (13.5 × 7 × 5.5 cm) and proofed in an incubator (Siam Incubator Systems Co., Ltd., Bangkok, Thailand) at 40 °C with 80% relative humidity for 40 min. Thereafter, the dough was baked in an electric oven (HOM-23J402, Homemate, Pathum Thani, Thailand) at 190 °C for 30 min and was glazed with beaten egg yolk in the middle of baking. The bread was depanned, cooled, weighed, packaged in a sealed oriented polypropylene bag and stored at room temperature until analyses on the next day. Bread samples were prepared in two experimental replications, and six loaves of bread were baked for each treatment within each replication.

### 2.5. Bread Analyses

#### 2.5.1. Physical and Chemical Characteristics

Loaf volume (cm^3^) was determined by the rapeseed displacement method according to the AACC method 10–05.01 [28] with a slight modification, using white sesame seeds instead of rapeseeds. Specific volume was calculated as loaf volume (cm^3^) divided by loaf weight (g). 

Bread crumb samples taken from the center of the bread loaf were analyzed for moisture content (%), water activity and color. Moisture content and water activity (a_w_) were determined using the AOAC method 10.1.2 [26] and a water activity meter (LabMaster-aw, Novasina, Schwyz, Switzerland), respectively. Color values (CIE L*, a* and b*) were measured by a colorimeter (Ultrascan, Hunterlab, Reston, VA, USA) at a 10° observer angle using D65 as a light source. All measurements were performed in triplicate for each experimental replication.

Texture profile analysis (TPA) was performed to determine crumb hardness (N), cohesiveness, gumminess (N), chewiness (N), springiness and resilience using a texture analyzer (TA-XT Plus, Stable Micro Systems, Surrey, UK) equipped with a P/36R stainless steel probe. The bread loaf was cut into slices (2 cm thickness) and three slices taken from the center of the loaf were cut into cubes (2 × 2 × 2 cm) [29]. The measurements were carried out at constant speeds of 2 mm/s for pre-test and 3 mm/s for test and post-test, with a trigger force of 5 g to compress the crumb at 40% deformation and with 5 s between each stroke. The cutting forces (N) of all bread crumb samples were measured using a blade set with a knife edge (HDP/BS) at constant speeds of 0.5 mm/s for pre-test, 3 mm/s for test and 10 mm/s for post-test, with a trigger force of 5 g [30]. Twelve measurements were taken from the same sample within each experimental replication.

#### 2.5.2. Sensory Characteristics

Bread crumb samples for sensory testing were prepared similarly as described for instrumental texture analysis. A traditional descriptive test, according to a profile method adapted from Keane [31] and used in other studies [32,33,34], was conducted to evaluate the sensory characteristics of the bread crumb samples. Nine trained descriptive panelists (all females, age range 39–57 y) affiliated with Kasetsart University Sensory and Consumer Research (KUSCR) Center (Bangkok, Thailand) participated in the test. The number of panelists used in this study fell within the range of 8–12 as suggested by Heymann et al. [35] for descriptive analysis. The panel completed 120 h of descriptive training in identifying and quantifying sensory attributes of various food categories, and had more than 2000 h of testing experiences with a variety of food products including bread. 

The procedure of the descriptive test involved using a trained panel to develop a sensory lexicon, which took three 3 h sessions in this study. Panelists individually evaluated bread crumb samples and identified terms for describing the flavor, texture and aftertaste characteristics of the samples. Then, they discussed possible terms, and compiled a list of attributes for testing. The panel also discussed and reached agreement on attribute definitions, references, reference intensities and evaluation procedures. Subsequently, the process continued with two 3 h training sessions in which the panelists practiced scoring each attribute on a 15 cm line scale, with 0 meaning “none” and 15 meaning “extremely high”. 

Thereafter, product testing was performed in five 3 h sessions. Two replications were evaluated for each of the seven bread crumb samples and serving order was randomized within each replication. Each panelist received 4 pieces (2 cm cube) of each sample in a plastic cup covered with a lid and labeled with a three-digit blinding code. After tasting the sample, the panelists rated the intensities of all attributes on the 15 cm line scales. References were provided during evaluations to anchor values on the scales. Reverse osmosis deionized water was provided for panelists to cleanse their palates between samples. The panelists had at least a 10 min break after each sample evaluation. 

All evaluations were conducted in the sensory facility at KUSCR. The rooms were air conditioned (25 °C), had appropriate lighting and had no extraneous odors. The testing room was separated from the sample preparation room.

### 2.6. Statistical Analysis

Analysis of variance (ANOVA) was performed to determine differences among samples. For physical and chemical data, the effects of sample and replication were included in the ANOVA model. For sensory data, the effects of sample, replication and panelist, and an interaction effect between sample and panelist, were included in the ANOVA model. Duncan’s multiple range test (DMRT) was then used in the mean comparisons. Correlation analysis was conducted to investigate relationships among the quality parameters of flour and bread samples. A significance level of 0.05 was adopted in this research. Principal component analysis (PCA) with varimax rotation was performed to uncover relationships between sensory attributes and bread samples. Hierarchical cluster analysis (HCA) was conducted using Ward’s minimum variance method to classify bread samples into subgroups with similar sensory characteristics. Non-significant attributes (*p* > 0.05) were excluded from the data set before performing PCA and HCA. The statistical software used for ANOVA, DMRT and correlation analysis was IBM SPSS Statistics version 28.0 (Thaisoftup Co., Ltd., Bangkok, Thailand), and that for PCA and HCA was XLSTAT version 19.6 (Addinsoft, New York, NY, USA).

## 3. Results and Discussion

### 3.1. Flour Characteristics

The sorghum and rice flours had slightly lower (*p* ≤ 0.05) moisture contents than tapioca flour and corn starch (Table 2). As a result, the moisture contents of flours T1–T7, used for breadmaking, tended to decrease with increased proportions of sorghum flour in the blends. However, the differences were marginal. 

Sorghum flour had a darker color than other single flours/starch. It had the lowest L* (lightness) value and the highest a* (redness) and b* (yellowness) values (Table 2). Although KU 804 is a white sorghum cultivar, the grains’ pericarp and endosperm are light brown. Thus, the darker color of the sorghum flour was attributed to the color of sorghum grains. In addition, tannins and polyphenol oxidase activity in sorghum grains could accelerate enzymatic browning and have an impact on the color of sorghum flour [36]. For composite flour samples, their color values were affected by the proportions of sorghum flour present in the blends. As per Table 2, the L* values of flours T1–T7 decreased (*p* ≤ 0.05), while a* and b* values increased (*p* ≤ 0.05), with each increase in sorghum flour level. 

For pasting properties, the results show that pasting temperature and peak time were the highest (*p* ≤ 0.05) for rice flour, followed by sorghum flour, corn starch and tapioca flour, respectively (Table 3). The higher pasting temperature and peak time indicated, the higher the heat energy required to alter the crystalline structure of starch granules to trigger gelatinization [12]. Similar findings were reported by Onyango et al. [6]. Peak viscosity, which indicates water-binding capacity [37], was the lowest (*p* ≤ 0.05) for sorghum flour, followed by rice flour, corn starch and tapioca flour, respectively. According to Akin and Miller [18], sorghum starch granules are embedded tightly in a protein matrix, which restricts their ability to absorb water, thus hindered gelatinization and resulting in high gelatinization temperature. In addition, the fiber in whole sorghum grain flour could compete with starch granules in the swelling process, thereby restricting starch gelatinization [2]. After gelatinization with continuous stirring, the decrease in viscosity (breakdown viscosity) was the lowest (*p* ≤ 0.05) for sorghum flour as compared to other single flours/starch. This indicates that sorghum flour paste was the most stable under heat and high shear conditions [12]. Upon cooling, the final and setback viscosities were higher (*p* ≤ 0.05) for sorghum and rice flours than for tapioca flour and corn starch. The higher final and setback viscosities indicate higher starch retrogradation and syneresis rates, which could adversely affect the texture, especially the softness, of bread after baking and during storage [12].

The pasting properties of flours T1–T7 used for breadmaking varied (*p* ≤ 0.05) depending on the proportions of sorghum flour in the blends (Table 3). The peak time was slightly higher (*p* ≤ 0.05) for flours with sorghum (T2–T7) than without (T1). The pasting temperature increased (*p* ≤ 0.05), while peak and breakdown viscosities decreased (*p* ≤ 0.05), with increased sorghum flour. Setback viscosity varied slightly among composite flours with 0–40% sorghum flour (T1–T4). As the amount of sorghum flour in the blends increased to 70% (T5) and 85% (T6), the setback viscosity of the blends increased dramatically (*p* ≤ 0.05). Composite flours T5 and T6 even had higher setback viscosities than pure sorghum flour (T7), and the results could be attributed to the interaction of sorghum flour with other flours in the blends, especially rice flour.

### 3.2. Bread Characteristics

#### 3.2.1. Physical and Chemical Characteristics

**Figure 1:**
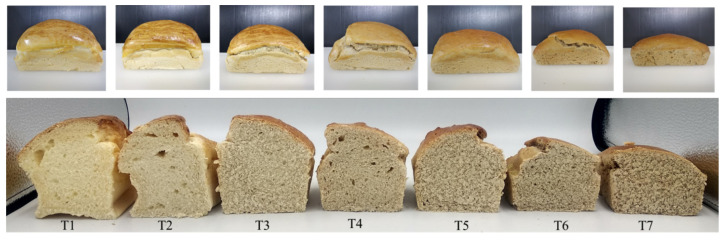
Breads prepared from different gluten-free composite flours. Formulations of composite flours T1–T7 are shown in Table 1.

The loaf specific volume decreased (*p* ≤ 0.05) with increased sorghum flour, particularly at 70% levels and onwards (Table 4). Monthe et al. [2] reported similar findings for GF bread prepared from fermented cassava, sweet potato and sorghum flour blends. The decrease in bread specific volume observed in the present study was probably influenced by the starch, protein and fiber components of sorghum flour. According to Yano [11], gas bubbles formed during mixing and fermentation are covered by starch granules in a GF dough system. Therefore, starch gelatinization contributes to the development of a cohesive crumb network that traps gas bubbles and prevents the loss of carbon dioxide and crust collapse [38]. The delay in starch gelatinization and the reduction in starch viscosity with increased sorghum flour in the blends (Table 3) could lead to a decrease in the gas retention ability of GF dough, and hence a decrease in loaf specific volume [37,39,40]. This was supported by the correlation results, which showed the specific volume being negatively correlated with pasting temperature (Pearson correlation coefficient (r) = −0.897; *p* ≤ 0.05), but positively correlated with peak viscosity (r = 0.754; *p* ≤ 0.05). Additionally, sorghum proteins, specifically kafirin, were hydrophobic and lacked the ability to form a cohesive, gas-holding viscoelastic dough, because their polypeptide chains were too short and too tightly cross-linked together [6,23]. These tended to aggregate in the dough’s liquid phase during baking, forming strands and lumps that disrupted starch gel [41]. Therefore, increasing sorghum flour in the blends increased dough hydrophobicity, thereby restricting the starch gelatinization process, which led to a decrease in bread specific volume. Moreover, the presence of endosperm and bran particles in whole grain sorghum flour could disrupt the uniformity of the starch gel and interfere with the formation of liquid films around gas cells [6], resulting in bread with a reduced volume and dense crumb structure, as seen in Figure 1. 

It should also be noticed that GF breads prepared from composite flours with 0 and 10% sorghum flour (T1 and T2 in Figure 1, respectively) contained large air pockets, known as alveoli, in the crumb. The formation of these alveoli was probably a consequence of fermentation and baking, during which carbon dioxide gas, a by-product of yeast fermentation, diffused into the dough’s air cells formed during mixing, causing them to expand during proofing and baking [42]. Based on the RVA results (Table 3), the peak viscosities of composite flours T1 and T2 were high, thus the doughs could trap the gas and expand to a great extent, resulting in large alveoli formation. On the contrary, the GF bread prepared from a composite flour with 25% sorghum flour (T3) did not contain large alveoli, while having a good volume with uniform and evenly distributed air cells. This was probably because the viscosity of T3 dough was at an optimal level to allow the dough to expand and create a good crumb structure without alveoli formation. The alveoli were observed again in bread with 40% sorghum flour (T4). Given that the peak viscosity of composite flour T4 was low, the formation of alveoli in the T4 bread could be due to the coalescence of small air cells during proofing and baking. As the sorghum flour levels exceeded 40%, dough viscosities decreased to a greater extent, leading to the collapse of dough and resulting in bread with a very dense crumb structure, without large alveoli formation. 

Regarding color, increasing the sorghum flour levels in the blends reduced (*p* ≤ 0.05) lightness (L*) and yellowness (b*), while increasing (*p* ≤ 0.05) redness (a*), in the bread crumb (Table 4). As per Figure 1, bread crumb samples with higher sorghum flour levels became darker in color. Nieto-Mazzocco et al. [43] reported consistent findings when sorghum flour was used in combination with amaranth flour. De Aguiar et al. [13] attributed the intense crumb color of sorghum bread to pigments in the sorghum pericarp. According to Jafari et al. [44], the bread crumb color was mainly affected by the color of flour being used for breadmaking, rather than the color pigments derived from Maillard and caramelization reactions. The correlation results also revealed significant relationships (*p* ≤ 0.05) between color values of flour blends and those of bread crumb samples (r = 0.992, 0.923 and −0.937 for L*, a* and b*, respectively). Consumers in certain countries such as Germany and Eastern Europe associated dark crumb color with healthiness [45]. Thus, the darker crumb color resulting from sorghum flour could be beneficial for the healthy product market [46]. In recent decades, consumers have shown a growing interest in incorporating black and colored breads into their diets due to an awareness of the benefits from antioxidants and bioactive compounds [47].

The moisture content and water activity (a_w_) of all GF bread samples varied in a narrow range of 43.00–44.65% and 0.880–0.894, respectively (Table 4). Statistical analysis revealed that the amount of sorghum flour in flour blends had no significant (*p* > 0.05) effect on moisture content and a_w_ of breads. 

Table 5 shows the results of the instrumental texture analysis of bread crumb samples. Significant differences (*p* ≤ 0.05) were found for almost all parameters except gumminess. Hardness tended to increase with increased sorghum flour, particularly at 40% levels onwards. The increase in hardness corresponded to the decrease in bread specific volume (Table 4) and the structure of the bread crumb, which became denser at higher sorghum flour levels (Figure 1). Crumb hardness was negatively correlated with bread specific volume (r = −0.917; *p* ≤ 0.05). Other studies [48,49] also reported similar relationships between the two parameters.

Unlike hardness, cohesiveness and cutting force decreased (*p* ≤ 0.05) as the proportions of sorghum flour increased (Table 5). Cohesiveness refers to the internal bond strength of the bread crumb and reflects its internal cohesion [2,6], while cutting force represents the force required to cut through the bread crumb [30]. Thus, the results indicate that the bread crumb became more prone to breaking apart and crumbling more easily with increased sorghum flour. Such changes could be attributed to the high degree of relative crystallinity of sorghum starch granules, as explained by Torbica et al. [10]. The cohesiveness of the bread crumb was likely influenced by the setback viscosity of flour being used for bread making. According to Marti et al. [50], a low setback value indicates a low rate of starch retrogradation and syneresis, which contribute to maintaining softness and cohesiveness in the bread crumb after baking and during storage. In this study, the setback viscosity of flour blends tended to increase with increased proportions of sorghum flour (Table 3), leading to an increase in starch retrogradation. As a result, the cohesiveness of the bread crumb decreased due to increased starch retrogradation with increased sorghum flour levels. This is supported by the correlation results, which show negative relationship between crumb cohesiveness and setback viscosity in flour blends (r = −0.815; *p* ≤ 0.05). According to De Alcântara et al. [51], the decreases in cohesiveness and cutting force of bread crumb could also be attributed to the higher contents of fiber and/or resistant starch as sorghum flour levels increased. These components influence water absorption, thermal properties, and starch gelatinization, resulting in a weak disintegrating crumb. In addition to the decrease in cohesiveness and cutting force, the chewiness, springiness and resilience of bread crumb also decreased with increased sorghum flour levels (Table 5), indicating a reduction in the elasticity of bread crumb [52]. This is because sorghum proteins lack the ability to form a viscoelastic dough [6,23].

#### 3.2.2. Sensory Characteristics of Gluten-Free Bread

Table 6 shows a sensory lexicon consisting of 40 attributes for evaluating the flavor, texture and aftertaste characteristics of GF bread samples prepared from composite flours with varying sorghum flour levels. All attributes are provided with their definitions, evaluation procedures, references and reference intensities. The reference samples in the lexicon were selected from a wide variety of commercial food ingredients and food products following a long discussion process among all panelists based on the goodness of fit with the attributes and definitions. Well-defined and -referenced attributes facilitate accurate and precise communication regarding bread’s sensory characteristics across panelists [53]. 

The mean intensities of all 40 sensory attributes of GF breads with varying sorghum flour levels in flour blends are given in Table 7. ANOVA and DMRT results reveal that 27 out of the 40 attributes evaluated were different (*p* ≤ 0.05) among the samples. Large differences across the samples were detected for cohesiveness, springiness, moistness and graininess, suggesting the higher impact of sorghum flour on texture than on the flavor characteristics of GF breads.

**Figure 2:**
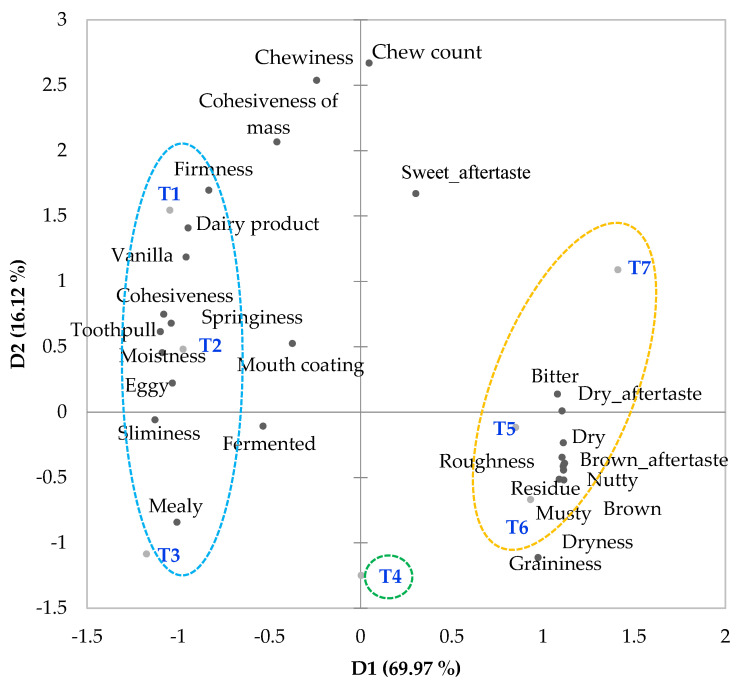
PCA biplot illustrating the relationships between sensory attributes and breads prepared from gluten-free composite flours with varying sorghum flour levels. Formulations of composite flours T1T7 are shown in Table 1. Circles illustrate the three sample clusters based on HCA.

The results from PCA indicate that significant attributes divided into two key dimensions explained 86.08% of total variability (69.97% and 16.12%, respectively), as shown in Figure 2. On PC1, heavily loaded attributes with absolute loading values greater than 0.6 [33] included brown, dry, musty, nutty, bitter, graininess, roughness, residue, dryness, brown aftertaste and dry aftertaste in the positive dimension, and dairy product, eggy, vanilla, firmness, cohesiveness, toothpull, springiness, moistness, sliminess and mealy in the negative dimension. On PC2, the heavily loaded attributes, including chewiness, cohesiveness of mass, chew count and sweet aftertaste, were all in the positive dimension. Based on HCA, the GF bread samples could be categorized into three clusters, as illustrated in the PCA biplot (Figure 2). Breads prepared from composite flours with 0–25% sorghum flour (T1, T2 and T3) were grouped together and formed cluster 1. The bread with 40% sorghum flour (T4) formed cluster two all by itself, while breads with 70–100% sorghum flour (T5, T6 and T7) were grouped together in cluster 3.

The breads in cluster 1 with 0–25% sorghum flour (T1, T2 and T3) were rated higher (*p* ≤ 0.05) in terms of dairy product flavor, cohesiveness, toothpull, and moistness, but lower (*p* ≤ 0.05) in terms of brown, dry and roughness than the rest of the samples (Table 7). In addition, the samples in this cluster tended to receive higher ratings in terms of eggy, fermented, vanilla, firmness, springiness, chewiness, cohesiveness of mass and mealy, yet lower ratings in terms of musty, nutty, bitter taste, sliminess, graininess, chew count, dryness, brown aftertaste and dry aftertaste than other samples. 

The bread with 40% sorghum flour (T4) was differentiated from the samples in cluster 1 by higher (*p* ≤ 0.05) intensities in brown, dry and roughness, but lower (*p* ≤ 0.05) intensities in dairy product, vanilla, cohesiveness, toothpull and moistness (Table 7). Additionally, T4 tended to be rated higher in musty and nutty, but lower in eggy and firmness than the samples in cluster 1. 

As the proportions of sorghum flour increased from 40% (T4) to 70–100% (T5, T6 and T7), further increases in brown, dry, nutty, bitter, graininess, brown aftertaste and dry aftertaste, along with decreases in cohesiveness, springiness and moistness of breads, were observed (Table 7). The levels of sorghum flour at which significant changes were observed as compared to T4 varied across attributes. For flavors, a significant change in the brown note was observed at 70% sorghum flour, while changes in dry, nutty and bitter taste were significant with 100% sorghum flour. For textures, significant changes in cohesiveness were detected at 70% sorghum flour, while changes in springiness and moistness became significant at 85% sorghum flour. 

The effects of sorghum flour on flavor characteristics—specifically musty, nutty and bitterness—have been documented in the literature. The increase in a musty note with increased sorghum flour levels could be linked to the volatile compounds present in sorghum grains, such as geosmin, 1,2-dimethoxybenzene, 3-octanone and 1,2,4-trimethoxybenzene [54], while the increase in a nutty note was probably caused by the proteins in sorghum grains, in relation to the Maillard reaction occurring during baking [55]. The phenolic compounds and tannins present in the outer layer of sorghum grains (pericarp and testa) were possibly the main contributors to a bitter taste [23,56]. It was discovered that the content of proanthocyanidins, the common form of condensed tannin responsible for bitterness and astringency in sorghum, was the lowest for sorghum grains with white pericarp [24,56]. This could justify the low bitterness intensities (less than 0.30 out of 15 scores) of all bread samples in this study, even the bread made with 100% sorghum flour. 

The decreases in cohesiveness, springiness and moistness and the increase in graininess of breads with increased sorghum flour levels could have adverse effects on consumer acceptance, because the consumers generally preferred sorghum bread with moist crumbs, while they disliked the grainy texture [13]. Other research reported similar effects of sorghum flour on the texture properties of bread. For instances, Sharanagat et al. [36] and Muggah et al. [57] reported that GF bread made from sorghum flour had an undesirably dry and crumbly texture. 

The current study also found significant relationships between texture properties that were evaluated by trained panelists and by a texture analyzer. For instance, sensory cohesiveness was positively correlated with instrumental cohesiveness (r = 0.976, *p* ≤ 0.05), while sensory springiness was positively correlated with instrumental springiness (r = 0.978, *p* ≤ 0.05) and resilience (r = 0.956, *p* ≤ 0.05). Sensory firmness and cohesiveness were in alignment with instrumental cutting force (r = 0.879, *p* ≤ 0.05 and 0.932, *p* ≤ 0.05, respectively). Therefore, instrumental analysis could be used to evaluate these texture attributes in place of sensory analysis when expert panelists are not available. However, inconsistent results were observed between sensory and instrumental hardness values. A lack of relationship between the two data could be attributed to several reasons, one of which was the misleading similarity in language used by panelists and instrumentalists. They may use the same words but measure different properties [58]. 

## 4. Conclusions

White sorghum flour could be used in combination with rice flour, tapioca flour and corn starch to make GF bread. Increasing sorghum flour increased color intensity, pasting temperature and setback viscosity, while decreasing the peak and breakdown viscosities of the flour blends. For GF bread, increasing sorghum flour levels led to decreases in specific volume, cohesiveness, springiness, chewiness and moistness, but increases in color intensity, brown and nutty flavors, graininess and roughness. Sorghum flour could be used in a flour blend at the maximum level of 25% to get a good bread volume without sacrificing its texture quality. Future studies should investigate the enhancement effects of different hydrocolloids and emulsifies to enable the use of sorghum flour in blends at levels higher that 25%. A consumer test should be carried out to determine the consumer acceptance of GF bread made with sorghum, rice and tapioca flours and corn starch. Information on the physicochemical properties (e.g., pasting properties, color values) of white sorghum flour can be useful for exploring its application in other GF products. The sensory lexicon developed in this research can be beneficial to future research on the sensory characteristics of GF bread. 

## Figures and Tables

**Table 1:** Formulations of composite gluten-free flours used for breadmaking in the study.

Composite Flours	Sorghum Flour (%)	Rice Flour (%)	Tapioca Flour (%)	Corn Starch (%)
T1	0	33.3	33.3	33.3
T2	10	30	30	30
T3	25	25	25	25
T4	40	20	20	20
T5	70	10	10	10
T6	85	5	5	5
T7	100	0	0	0

**Table 2:** Moisture content and color values (means ± standard deviations) of single flours/starch and composite flours used in the study.

	Moisture Content (%)	Color Values		
		L*	a*	b*
**Single flours/starch**				
Sorghum flour	12.27 ± 0.11 ^B^	80.95 ± 0.56 ^C^	1.67 ± 0.01 ^A^	12.27 ± 0.08 ^A^
Rice flour	12.17 ± 0.11 ^B^	91.99 ± 0.02 ^B^	−0.24 ± 0.02 ^C^	3.60 ± 0.02 ^C^
Tapioca flour	12.76 ± 0.19 ^A^	92.83 ± 0.10 ^A^	0.10 ± 0.03 ^B^	2.80 ± 0.01 ^D^
Corn starch	12.57 ± 0.14 ^A^	93.41 ± 0.07 ^A^	−0.34 ± 0.02 ^D^	4.80 ± 0.08 ^B^
**Composite flours ^#^**				
T1 (0%)	12.52 ± 0.12 ^a^	92.26 ± 0.06 ^a^	−0.17 ± 0.05 ^g^	3.44 ± 0.09 ^g^
T2 (10%)	12.44 ± 0.19 ^a^	90.87 ± 0.46 ^b^	0.03 ± 0.03 ^f^	4.61 ± 0.00 ^f^
T3 (25%)	12.46 ± 0.14 ^a^	89.19 ± 0.21 ^c^	0.21 ± 0.04 ^e^	5.61 ± 0.11 ^e^
T4 (40%)	12.50 ± 0.17 ^a^	87.22 ± 0.69 ^d^	0.44 ± 0.12 ^d^	7.01 ± 0.20 ^d^
T5 (70%)	12.40 ± 0.13 ^ab^	83.20 ± 0.41 ^e^	1.05 ± 0.02 ^c^	9.88 ± 0.31 ^c^
T6 (85%)	12.32 ± 0.10 ^b^	82.03 ± 0.12 ^f^	1.24 ± 0.05 ^b^	10.42 ± 0.02 ^b^
T7 (100%)	12.27 ± 0.11 ^b^	80.95 ± 0.56 ^f^	1.67 ± 0.01 ^a^	12.27 ± 0.08 ^a^

^#^ Formulations of composite flours T1–T7 are shown in Table 1. The numbers in brackets indicate the percentages of sorghum flour in flour blends. ^A–D^ Means of single flours/starch with different letters are significantly different (*p* ≤ 0.05). ^a–g^ Means of composite flours with different letters are significantly different (*p* ≤ 0.05).

**Table 3:** Pasting properties (means ± standard deviations) of single flours/starch and composite flours used in the study.

	Pasting Temperature (°C)	Peak Time (min)	Peak Viscosity (cp)	Breakdown Viscosity (cp)	Setback Viscosity (cp)	Final Viscosity (cp)
**Single flours/starch**						
Sorghum flour	87.4 ± 0.2 ^B^	6.0 ± 0.1 ^B^	1498.5 ± 27.6 ^D^	69.3 ± 7.5 ^D^	1190.0 ± 66.0 ^A^	2619.2 ± 30.9 ^B^
Rice flour	90.5 ± 0.6 ^A^	6.8 ± 0.1 ^A^	2156.0 ± 36.3 ^C^	376.8 ± 52.6 ^C^	1191.8 ± 6.8 ^A^	2993.8 ± 63.4 ^A^
Tapioca flour	65.1 ± 0.1 ^D^	3.8 ± 0.0 ^D^	4335.2 ± 96.4 ^A^	2843.2 ± 93.1 ^A^	686.3 ± 6.8 ^B^	2198.3 ± 6.6 ^C^
Corn starch	76.6 ± 0.2 ^C^	5.2 ± 0.0 ^C^	2525.2 ± 32.8 ^B^	931.7 ± 10.4 ^B^	629.5 ± 18.4 ^B^	2223.0 ± 26.4 ^C^
**Composite flours ^#^**						
T1 (0%)	71.5 ± 0.4 ^f^	5.8 ± 0.0 ^c^	2757.7 ± 12.7 ^a^	670.8 ± 27.6 ^a^	706.7 ± 20.3 ^c^	2793.5 ± 5.4 ^b^
T2 (10%)	72.8 ± 0.9 ^ef^	6.0 ± 0.1 ^b^	2394.3 ± 86.3 ^b^	523.0 ± 81.1 ^b^	606.3 ± 99.4 ^cd^	2607.0 ± 89.1 ^c^
T3 (25%)	73.4 ± 0.0 ^e^	6.1 ± 0.0 ^ab^	2011.5 ± 21.9 ^c^	374.3 ± 14.6 ^c^	542.8 ± 21.0 ^cd^	2180.0 ± 15.6 ^d^
T4 (40%)	75.4 ± 1.0 ^d^	6.1 ± 0.2 ^ab^	1827.6 ± 94.6 ^d^	353.8 ± 54.0 ^c^	504.7 ± 82.5 ^d^	1996.0 ± 21.7 ^e^
T5 (70%)	83.6 ± 0.4 ^c^	6.2 ± 0.1 ^a^	1737.6 ± 75.1 ^de^	217.2 ± 1.6 ^d^	1653.5 ± 67.8 ^a^	3219.7 ± 77.3 ^a^
T6 (85%)	86.0 ± 0.4 ^b^	6.2 ± 0.1 ^a^	1589.0 ± 84.9 ^ef^	102.0 ± 29.2 ^e^	1786.5 ± 80.8 ^a^	3157.7 ± 75.9 ^a^
T7 (100%)	87.4 ± 0.2 ^a^	6.0 ± 0.1 ^b^	1498.5 ± 27.6 ^f^	69.3 ± 7.5 ^e^	1190.0 ± 66.0 ^b^	2619.2 ± 30.9 ^c^

^#^ Formulations of composite flours T1–T7 are shown in Table 1. The numbers in brackets indicate the percentages of sorghum flour in flour blends. ^A–D^ Means of single flours/starch with different letters are significantly different (*p* ≤ 0.05). ^a–f^ Means of composite flours with different letters are significantly different (*p* ≤ 0.05).

**Table 4:** Loaf specific volume, color values, moisture content and water activity (means ± standard deviations) of breads prepared from gluten-free composite flours of varying sorghum flour levels.

Composite Flours ^#^	Loaf Specific Volume (cm^3^/g)	Crumb Color			Moisture (%) ^ns^	Water Activity (a_w_) ^ns^
		L*	a*	b*		
T1 (0%)	3.24 ± 0.01 ^a^	80.26 ± 1.68 ^a^	3.16 ± 0.32 ^d^	23.32 ± 0.19 ^a^	44.23 ± 0.81	0.894 ± 0.001
T2 (10%)	3.17 ± 0.01 ^b^	79.20 ± 2.02 ^ab^	3.09 ± 0.11 ^d^	21.73 ± 1.88 ^ab^	43.00 ± 0.59	0.892 ± 0.002
T3 (25%)	3.15 ± 0.00 ^b^	75.67 ± 2.10 ^bc^	3.70 ± 0.25 ^c^	21.20 ± 2.33 ^bc^	43.37 ± 0.71	0.894 ± 0.002
T4 (40%)	3.11 ± 0.03 ^b^	73.24 ± 0.87 ^cd^	4.10 ± 0.13 ^b^	20.46 ± 0.75 ^bcd^	43.52 ± 0.99	0.892 ± 0.002
T5 (70%)	2.95 ± 0.02 ^c^	69.91 ± 0.66 ^de^	4.40 ± 0.41 ^a^	20.37 ± 0.44 ^bcd^	43.68 ± 0.68	0.891 ± 0.000
T6 (85%)	2.53 ± 0.02 ^d^	68.36 ± 2.65 ^e^	4.32 ± 0.19 ^ab^	19.61 ± 1.68 ^cd^	44.65 ± 0.82	0.893 ± 0.003
T7 (100%)	2.11 ± 0.04 ^e^	66.20 ± 4.63 ^e^	4.57 ± 0.27 ^a^	18.95 ± 1.05 ^d^	44.44 ± 0.85	0.880 ± 0.018

^#^ Formulations of composite flours T1–T7 are shown in Table 1. The numbers in brackets indicate the percentages of sorghum flour in flour blends. ^a–e^ Means within column with different letters are significantly different (*p* ≤ 0.05). ^ns^ Means within column are not significantly different (*p* > 0.05).

**Table 5:** Instrumental texture parameters (means ± standard deviations) of breads prepared from gluten-free composite flours with varying sorghum flour levels.

Composite Flours ^#^	Hardness (N)	Cohesiveness	Gumminess (N) ^ns^	Chewiness (N)	Springiness	Resilience	Cutting Force (N)
T1 (0%)	6.52 ± 1.84 ^e^	0.66 ± 0.03 ^a^	5.03 ± 0.64	4.83 ± 0.48 ^a^	0.95 ± 0.02 ^a^	0.34 ± 0.02 ^a^	17.23 ± 0.12 ^a^
T2 (10%)	8.77 ± 3.70 ^de^	0.55 ± 0.01 ^b^	4.72 ± 1.02	4.20 ± 1.10 ^ab^	0.91 ± 0.03 ^ab^	0.26 ± 0.01 ^b^	11.34 ± 0.33 ^b^
T3 (25%)	7.31 ± 0.95 ^e^	0.48 ± 0.05 ^bc^	4.08 ± 0.61	3.41 ± 0.15 ^abc^	0.88 ± 0.04 ^abc^	0.20 ± 0.05 ^bc^	9.84 ± 0.13 ^c^
T4 (40%)	12.36 ± 3.47 ^cd^	0.40 ± 0.04 ^cd^	4.85 ± 0.86	3.65 ± 0.15 ^abc^	0.78 ± 0.14 ^bcd^	0.16 ± 0.03 ^cd^	5.91 ± 0.07 ^d^
T5 (70%)	14.87 ± 2.97 ^bc^	0.31 ± 0.06 ^de^	4.25 ± 0.69	2.82 ± 0.64 ^bc^	0.74 ± 0.04 ^cd^	0.13 ± 0.04 ^d^	4.67 ± 0.03 ^e^
T6 (85%)	18.17 ± 2.29 ^ab^	0.26 ± 0.02 ^e^	4.04 ± 0.32	2.50 ± 0.35 ^c^	0.65 ± 0.12 ^d^	0.11 ± 0.02 ^d^	4.63 ± 0.16 ^e^
T7 (100%)	20.22 ± 0.48 ^a^	0.27 ± 0.03 ^e^	5.13 ± 0.04	3.21 ± 0.42 ^bc^	0.64 ± 0.04 ^d^	0.11 ± 0.01 ^d^	4.87 ± 0.54 ^e^

^#^ Formulations of composite flours T1–T7 are shown in Table 1. The numbers in brackets indicate the percentages of sorghum flour in flour blends. ^a–e^ Means within columns with different letters are significantly different (*p* ≤ 0.05). ^ns^ Means within columns are not significantly different (*p* > 0.05).

**Table 6:** Attributes, definitions, evaluation procedure, references and reference intensities for evaluating sensory characteristics of gluten-free breads.

Attributes	Definitions and Evaluation Procedure	References and Reference Intensities
***Flavor***		
Baked flour	A flavor of baked flour which consists of cereal, dry, sweet and brown notes.	Ritz crackers (original) (1 piece) = 5.0 Jacob’s cream cracker (original) (1/4 piece) = 9.0
Baking soda	A flavor associated with baking soda.	McGarrett baking soda 0.25 g in 100 ml water = 2.0 0.50 g in 100 ml water = 5.5
Brown	A full, round flavor impression always characterized as some degree of darkness, generally associated with other attributes such as baked, roasted, sweet, etc.	S&W canned pinto beans = 5.0
Dairy product	A sweet and milky flavor associated with dairy products.	Meji full fat pasteurized milk = 8.5
Dry	A dry flavor of food products achieved from drying or dehydration process.	Doi Kham soya milk powder 2 g in 400 ml water = 3.0 Soya milk powder (pure) = 7.0
Eggy	A flavor associated with cooked whole egg with a mild sulfur note.	Peng Kee crinkle cookie (2 × 2 cm piece) = 5.0
Fermented	A sour, pungent and slightly sweet flavor associated with fermented starches, grains, vegetables, or fruits.	Kewpie 4.2% Hom Mali Rice vinegar 2 mL in 250 mL water = 2.5
Musty	A flavor associated with stale products or closed air space/poor ventilation area.	Kellogg’s All-Bran (original) 3 pieces = 3.5, 6 pieces = 7.0
Nutty	A sweet, slightly roasted, dry, woody flavor associated with nuts, wheat germ and certain whole grains.	Dr. Green wheat germ = 7.5
Sweet aromatic	A flavor associated with the impression of all sweet substances.	Mitr Phol brown sugar 40 g in 400 mL water = 3.5 Brown sugar (pure) = 5.5
Vanilla	A flavor associated with vanilla consisting of brown, sweet and dry bark notes.	Winner vanilla flavor 0.3 mL in 200 mL water = 4.0
Sweet	A fundamental taste sensation of which sucrose is typical.	20 g/L sucrose solution = 2.0 50 g/L sucrose solution = 5.0
Salty	A fundamental taste sensation of which sodium chloride is typical.	2 g/L NaCl solution = 2.5 3.5 g/L NaCl solution = 5.0
Bitter	A fundamental taste sensation of which caffeine is typical.	0.1 g/L caffeine solution = 2.0 0.35 g/L caffeine solution = 3.5
Astringent	The complex of drying, puckering and shrinking sensations in the mouth.	0.35 g/L alum solution = 1.5
Tooth-etch	A drying/dragging sensation perceived when the tongue is rubbing on the tooth surface.rubbing on the tooth surface.	1 g/L alum solution = 4.0
***Texture***		
Hardness	Force required to compress the sample between molars to attain sample deformation.	S&P butter cake (0.5-in cube) = 3.0
Firmness	Resistance to compression of the sample with the first bite with molars.	S&P butter cake (0.5-in cube) = 3.0
Cohesiveness	The degree to which the sample deforms rather than crumbles or breaks on the first bite with molars.	S&P butter cake (0.5-in cube) = 7.0
Tooth pull	The force required to pull molars out of the sample.	Hershey’s creamy milk chocolate (1 piece) = 2.0
Springiness	The degree to which the sample returns to its original shape after compression with molars.	S&P butter cake (0.5-in cube) = 2.5
Chewiness	Difficulty in chewing the sample. Evaluate during 1–5 chews with molars.	Markenburg Marshies choco-vanilla (1/2 piece) = 4.0
Moistness	The amount of water perceived in the sample during chewing. Evaluate during 1–10 chews with molars.	S&P butter cake (0.5-in cube) = 7.0
Cohesiveness of mass	The degree to which the chewed sample holds together in mass. Evaluate after 15 chews with molars.	Lay’s Stax original flavored potato chips (1/2 piece) = 3.0 Ritz cracker (original) (1/2 piece) = 7.0
Sliminess	The degree to which a slimy, slippery, slightly viscous and soft gel-like sensation is perceived in the mouth. Evaluate after 15 chews with molars.	Doi Kham tomato Juice = 2.0
Graininess	The amount of round and small particles perceived in the mouth. Evaluate after 15 chews with molars.	Sunbites multigrain snack (1 piece) = 7.0
Mealy	The sensation of fine, soft, and somewhat rounded smooth and evenly distributed particles perceived when eating cooked starchy tubers such as potatoes, taro and yams. This attribute is perceived as the product is broken down during chewing. It is a geometrical attribute within the product itself and is not created by the chewing process. Evaluate after 15 chews with molars.	McGarrett mashed potato 10 g mashed potato, stir in 100 mL hot water, then put in microwave (1000 watt) for 1 min = 8.0
Roughness	The degree of abrasion of the chewed sample. Chew the sample with molars 15 times. Use the tip of the tongue to push the sample against the palate, then evaluate roughness of chewed sample.	S&P butter cake (0.5-in cube) = 2.0 Lay’s Stax original flavored potato chips (1/2 piece) = 6.0
Chew count	The number of times the sample is chewed until it is ready to swallow. Chew the sample with molars at a constant rate of 1 chew/s.	S&P butter cake (0.5-in cube) = 6.0
Mouth coating	A feeling of starchy or fatty coating in the mouth perceived immediately after swallowing the sample.	Meji full fat pasteurized milk = 6.0
Tooth pack	The amount of sample attached to teeth perceived immediately after swallowing the sample.	Lay’s Stax original flavored potato chips (1/2 piece) = 3.0 Thong Garden salted peanuts (2 halves) = 5.0
Residue	The amount of sample left in the mouth, except the tooth area, perceived immediately after swallowing the sample.	Lay’s Stax original flavored potato chips (1/2 piece) = 2.5 Thong Garden salted peanuts (2 halves) = 5.0
Dryness	The feeling of dryness perceived in the mouth and throat immediately after swallowing the sample.	Mitr Phol brown sugar = 4.0
***Aftertaste*** (after swallowing the sample for 30 s)		
Baked flour	The perception of baked flour flavor after swallowing.	
Brown	The perception of brown flavor after swallowing.	
Dairy product	The perception of dairy product flavor after swallowing.	
Dry	The perception of dry flavor after swallowing.	
Sweet aromatic	The perception of sweet aromatic after swallowing.	
Salty	The residual salty taste after swallowing.	
Sweet	The residual sweet taste after swallowing.	

**Table 7:** Sensory attribute intensities (means ± standard deviations) of breads prepared from gluten-free composite flours of varying sorghum flour levels.

Attributes	Composite Flours ^#^						
	T1 (0%)	T2 (10%)	T3 (25%)	T4 (40%)	T5 (70%)	T6 (85%)	T7 (100%)
**Flavor**							
Baked flour ^ns^	6.28 ± 0.26	6.21 ± 0.17	6.15 ± 0.26	6.03 ± 0.04	5.90 ± 0.30	6.04 ± 0.25	5.99 ± 0.06
Baking soda ^ns^	2.96 ± 0.06	2.87 ± 0.39	2.77 ± 0.05	2.77 ± 0.03	2.91 ± 0.02	2.96 ± 0.07	2.91 ± 0.24
Brown	2.83 ± 0.46 ^c^	3.02 ± 0.30 ^c^	2.97 ± 0.48 ^c^	3.58 ± 0.07 ^b^	3.88 ± 0.09 ^a^	4.04 ± 0.26 ^a^	4.01 ± 0.06 ^a^
Dairy product	3.43 ± 0.09 ^a^	3.21 ± 0.05 ^b^	3.21 ± 0.19 ^b^	2.84 ± 0.14 ^c^	2.86 ± 0.04 ^c^	2.78 ± 0.07 ^c^	2.93 ± 0.14 ^c^
Dry	2.39 ± 0.07 ^c^	2.40 ± 0.31 ^c^	2.47 ± 0.46 ^c^	2.92 ± 0.07 ^b^	3.06 ± 0.08 ^ab^	3.02 ± 0.13 ^ab^	3.34 ± 0.04 ^a^
Eggy	2.27 ± 0.01 ^ab^	2.33 ± 0.02 ^a^	2.41 ± 0.34 ^a^	2.03 ± 0.12 ^bc^	1.81 ± 0.04 ^c^	1.96 ± 0.06 ^c^	1.97 ± 0.04 ^c^
Fermented	1.53 ± 0.40 ^ab^	1.27 ± 0.08 ^c^	1.56 ± 0.28 ^a^	1.38 ± 0.15 ^bc^	1.34 ± 0.09 ^bc^	1.37 ± 0.06 ^bc^	1.37 ± 0.10 ^bc^
Musty	1.04 ± 0.29 ^c^	1.11 ± 0.07 ^bc^	1.05 ± 0.10 ^c^	1.34 ± 0.06 ^ab^	1.45 ± 0.04 ^a^	1.44 ± 0.10 ^a^	1.47 ± 0.04 ^a^
Nutty	2.75 ± 0.30 ^d^	2.89 ± 0.16 ^d^	2.95 ± 0.24 ^cd^	3.23 ± 0.30 ^bc^	3.44 ± 0.08 ^ab^	3.48 ± 0.02 ^ab^	3.64 ± 0.04 ^a^
Sweet aromatic ^ns^	2.51 ± 0.02	2.38 ± 0.00	2.59 ± 0.19	2.62 ± 0.02	2.68 ± 0.29	2.58 ± 0.16	2.70 ± 0.16
Vanilla	0.77 ± 0.01 ^a^	0.71 ± 0.25 ^ab^	0.72 ± 0.39 ^ab^	0.42 ± 0.04 ^c^	0.40 ± 0.02 ^c^	0.42 ± 0.04 ^c^	0.51 ± 0.17 ^bc^
Sweet ns	3.26 ± 0.24	3.19 ± 0.11	3.35 ± 0.41	3.19 ± 0.15	3.14 ± 0.04	3.15 ± 0.21	3.38 ± 0.14
Salty ns	4.77 ± 0.06	4.90 ± 0.30	4.37 ± 0.58	4.61 ± 0.08	4.63 ± 0.10	4.79 ± 0.29	4.79 ± 0.05
Bitter	0.00 ± 0.00 ^c^	0.02 ± 0.03 ^bc^	0.03 ± 0.04 ^bc^	0.08 ± 0.04 ^bc^	0.13 ± 0.18 ^abc^	0.19 ± 0.27 ^ab^	0.28 ± 0.08 ^a^
Astringent ^ns^	1.37 ± 0.05	1.20 ± 0.03	1.31 ± 0.10	1.26 ± 0.07	1.31 ± 0.06	1.34 ± 0.16	1.27 ± 0.04
Tooth-etch ^ns^	1.93 ± 0.05	1.78 ± 0.00	1.91 ± 0.18	1.84 ± 0.14	1.81 ± 0.00	1.86 ± 0.07	1.87 ± 0.16
**Texture**							
Hardness ^ns^	4.32 ± 0.11	4.21 ± 0.24	3.94 ± 0.42	3.83 ± 0.17	3.96 ± 0.06	4.04 ± 0.05	4.08 ± 0.07
Firmness	4.26 ± 0.17 ^a^	4.29 ± 0.15 ^a^	3.94 ± 0.15 ^ab^	3.58 ± 0.20 ^b^	3.74 ± 0.26 ^b^	3.63 ± 0.01 ^b^	3.78 ± 0.08 ^b^
Cohesiveness	10.34 ± 0.01 ^a^	9.64 ± 0.05 ^b^	8.98 ± 1.05 ^b^	7.98 ± 0.29 ^c^	6.71 ± 0.25 ^d^	6.19 ± 0.43 ^d^	6.39 ± 0.31 ^d^
Tooth pull	1.81 ± 0.18 ^a^	1.70 ± 0.03 ^a^	1.69 ± 0.07 ^a^	1.39 ± 0.16 ^b^	1.35 ± 0.07 ^b^	1.28 ± 0.03 ^b^	1.21 ± 0.13 ^b^
Springiness	3.43 ± 0.06 ^a^	3.21 ± 0.14 ^a^	2.83 ± 0.24 ^b^	2.69 ± 0.27 ^bc^	2.47 ± 0.20 ^cd^	2.17 ± 0.50 ^de^	1.99 ± 0.17 ^e^
Chewiness	4.25 ± 0.19 ^a^	4.19 ± 0.27 ^a^	3.76 ± 0.15 ^bc^	3.68 ± 0.26 ^c^	3.89 ± 0.44 ^abc^	3.63 ± 0.02 ^c^	4.18 ± 0.06 ^ab^
Moistness	5.46 ± 0.02 ^a^	5.44 ± 0.13 ^a^	5.44 ± 0.15 ^a^	4.92 ± 0.51 ^b^	4.47 ± 0.27 ^bc^	4.17 ± 0.16 ^c^	4.47 ± 0.20 ^bc^
Cohesiveness of mass	9.20 ± 0.50 ^a^	8.85 ± 0.55 ^ab^	8.55 ± 0.01 ^b^	8.75 ± 0.04 ^b^	8.57 ± 0.21 ^b^	8.59 ± 0.04 ^b^	8.78 ± 0.16 ^b^
Sliminess	2.94 ± 0.08 ^ab^	2.98 ± 0.16 ^a^	2.94 ± 0.01 ^ab^	2.82 ± 0.02 ^abc^	2.69 ± 0.01 ^abc^	2.63 ± 0.05 ^bc^	2.58 ± 0.23 ^c^
Graininess	2.87 ± 0.47 ^d^	3.12 ± 0.31 ^cd^	3.33 ± 0.00 ^c^	3.44 ± 0.16 ^bc^	3.69 ± 0.59 ^ab^	4.07 ± 0.02 ^a^	3.72 ± 0.47 ^ab^
Mealy	5.00 ± 0.16 ^bc^	5.16 ± 0.33 ^ab^	5.54 ± 0.16 ^a^	4.94 ± 0.24 ^bc^	4.77 ± 0.25 ^bc^	4.69 ± 0.04 ^c^	4.60 ± 0.17 ^c^
Roughness	3.19 ± 0.24 ^b^	3.21 ± 0.05 ^b^	3.24 ± 0.04 ^b^	3.58 ± 0.12 ^a^	3.72 ± 0.11 ^a^	3.69 ± 0.04 ^a^	3.82 ± 0.12 ^a^
Chew count	8.80 ± 0.05 ^a^	8.62 ± 0.10 ^ab^	8.21 ± 0.30 ^c^	8.31 ± 0.04 ^bc^	8.49 ± 0.16 ^abc^	8.39 ± 0.31 ^bc^	8.69 ± 0.03 ^ab^
Mouth coating	4.53 ± 0.43 ^abc^	4.88 ± 0.39 ^a^	4.84 ± 0.17 ^ab^	4.22 ± 0.47 ^c^	4.42 ± 0.20 ^bc^	4.64 ± 0.04 ^abc^	4.66 ± 0.16 ^abc^
Tooth pack ^ns^	1.39 ± 0.23	1.48 ± 0.08	1.52 ± 0.14	1.63 ± 0.13	1.57 ± 0.02	1.73 ± 0.30	1.64 ± 0.23
Residue	1.75 ± 0.08 ^c^	1.82 ± 0.19 ^bc^	1.86 ± 0.05 ^bc^	1.99 ± 0.06 ^abc^	2.08 ± 0.04 ^ab^	2.10 ± 0.09 ^ab^	2.22 ± 0.03 ^a^
Dryness	2.31 ± 0.03 ^c^	2.31 ± 0.00 ^c^	2.43 ± 0.22 ^bc^	2.64 ± 0.03 ^ab^	2.69 ± 0.07 ^ab^	2.73 ± 0.20 ^a^	2.87 ± 0.14 ^a^
**Aftertaste**							
Baked flour ^ns^	1.66 ± 0.05	1.64 ± 0.09	1.54 ± 0.03	1.58 ± 0.07	1.48 ± 0.06	1.44 ± 0.00	1.58 ± 0.16
Brown	1.01 ± 0.09 ^d^	1.11 ± 0.17 ^cd^	1.11 ± 0.23 ^cd^	1.33 ± 0.08 ^bc^	1.55 ± 0.02 ^ab^	1.49 ± 0.02 ^ab^	1.58 ± 0.08 ^a^
Dairy product ^ns^	1.28 ± 0.11	1.32 ± 0.06	1.29 ± 0.08	1.17 ± 0.03	1.26 ± 0.01	1.24 ± 0.02	1.25 ± 0.04
Dry	1.20 ± 0.07 ^b^	1.19 ± 0.19 ^b^	1.25 ± 0.14 ^b^	1.30 ± 0.08 ^b^	1.54 ± 0.17 ^a^	1.56 ± 0.05 ^a^	1.62 ± 0.05 ^a^
Sweet aromatic ^ns^	1.32 ± 0.06	1.29 ± 0.09	1.23 ± 0.01	1.39 ± 0.13	1.40 ± 0.14	1.47 ± 0.12	1.37 ± 0.10
Salty ^ns^	1.90 ± 0.06	1.89 ± 0.00	2.03 ± 0.17	2.11 ± 0.03	1.93 ± 0.06	1.97 ± 0.00	1.89 ± 0.10
Sweet	1.52 ± 0.01 ^a^	1.38 ± 0.02 ^ab^	1.46 ± 0.03 ^a^	1.31 ± 0.00 ^b^	1.49 ± 0.12 ^a^	1.45 ± 0.01 ^ab^	1.53 ± 0.29 ^a^

^#^ Formulations of composite flours T1–T7 are shown in Table 1. The numbers in brackets indicate the percentages of sorghum flour in flour blends. ^a–d^ Means within rows with different letters are significantly different (*p* ≤ 0.05). ^ns^ Means within rows are not significantly different (*p* > 0.05).

## Data Availability

The data in this study are available in the article.
